# Unraveling recurrent urinary tract infection in adulthood: a rare case report of unilateral partial duplex collecting system with ureterocele

**DOI:** 10.1097/MS9.0000000000001215

**Published:** 2023-09-01

**Authors:** Shailendra Katwal, Aastha Ghimire, Kusum Shrestha, Rochak Kansakar, Suban Amatya

**Affiliations:** a Department of Radiology, Dadeldhura Subregional Hospital, Dadeldhura; b Patan Academy of Health Sciences, Lalitpur; c Kist Medical College and Teaching Hospital, Nepal

**Keywords:** case report, CT urography, duplex collecting system, recurrent urinary tract infection, ureterocele

## Abstract

**Introduction:**

Duplication of the renal collecting system, known as the duplex collecting system, is a common congenital anomaly of the urinary tract. It can be partial or complete and affects 0.7–4% of the population, with a higher incidence in females. Ureteroceles are cystic dilations of the distal ureter and are often asymptomatic, particularly in adults.

**Case presentation:**

The authors present a case of a newly diagnosed partial duplex collecting system of the left kidney and left intravesical ureterocele, which was diagnosed for the first time at the age of 47 years, along with a history of symptoms suggestive of recurrent urinary tract infection and a urethral calculus which was surgically managed 5 years ago.

**Clinical discussion:**

The presence of a duplex collecting system can be observed even in males, with the possibility of recurrent urinary tract infection and the rare occurrence of an intravesical ureterocele. While ureteroceles are typically considered a congenital condition, they can also be diagnosed in adults.

**Conclusion:**

A partial duplex collecting system of the left kidney with left intravesical ureterocele in the age of 47 years in a male is a rare occurrence. Diagnosis and management of such urological cases can be challenging especially in a resource limited setting, which can be mitigated by awareness of unusual presentations, proper antenatal care, and access to proper diagnostic tools.

## Introduction

Highlights

- Duplex ureter is mostly diagnosed prenatally and during childhood, which along with ureterocele is rarely diagnosed in adults.
- Patients with prolonged duration of recurrent urinary symptoms should be evaluated for possible underlying structural abnormalities.
- Delayed diagnosis due to a lack of access to affordable diagnostic tests leads to prolonged symptoms in patients living in rural areas of countries like Nepal.

Duplication of the renal collecting system, also called as duplex collecting system, is one of the most common congenital anomaly of the urinary tract^1^. This anomaly can be either partial or complete, and is found in 0.7–4% of the world’s population, with a higher incidence in females compared to males^1^. The duplication is thought to be as a result of the duplication of the ureteric bud^2^. Most of the cases are asymptomatic uncomplicated duplication, while some may cause upper pole hydroureteronephrosis, secondary to ectopic insertion or ureterocele, and urinary tract infection (UTI), due to vesicoureteral reflux (VUR)^1^. Duplex collecting system is detected during the perinatal period or in pediatric patients, but in some rare cases, they may not be identified until adulthood^3^.

Ureteroceles are cystic dilation of the distal aspect of the ureter whose incidence is 1: 4000 people, occurring four times more often in women^4^. Ureteroceles, especially in adults, are generally asymptomatic and rarely diagnosed^5^. Detecting symptomatic ureterocele in adulthood is difficult and is often associated with secondary complications^6^. Here, we report the case of a 42-year-old male patient with a newly diagnosed partial duplex collecting system with ureterocele and a history of urethral calculus.

## Case details

A 47-year-old male presented to the Outpatient Department of our hospital with a complaint of experiencing a burning sensation during urination for the past 12 days. Additionally, he mentioned that urine dribbled out after he finished voiding, causing his undergarments to become soaked. He had no associated complains of increased urinary frequency, urgency, reddish discoloration of urine, fever, urinary incontinence, or flank pain. He had no history of diagnosed comorbidities like hypertension, diabetes mellitus, hyperlipidemia, or thyroid disease. However, the patient did mention experiencing recurrent urinary symptoms resembling the current episode for approximately the past 12 years.

**Figure 1:**
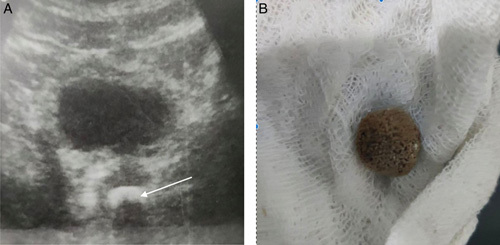
(A) Gray scale ultrasound image showing calculus in the prostatic urethra (white arrow). (B) Image of the urethral stone fragment after extraction.

The patient had an episode of severe lower abdominal pain and had previously undergone a cystoscopic litholapaxy procedure 5 years ago to remove calculus in the prostatic urethra. (Fig. 1A, B). He was advised for a computed tomography (CT) scan of the pelvis prior to the surgery but was unable to follow the recommendations due to his financial constraints. The patient underwent the procedure under spinal anesthesia. After proper positioning and ensuring anesthesia, the cystoscope was inserted and advanced into the prostatic urethra where the stone was visualized and fragmented and the fragments were flushed out by the use of saline irrigation. The postoperative course was uneventful.

The patient was born at home with no antenatal care visits attended by the mother. The patient vaguely recalled experiencing some urinary problems during childhood, but there were no documented records of those episodes. There was no known history of genital anomalies, difficulty in vision, developmental delay and behavioral disorders. No identifiable precipitating factor for the formation of renal calculus was found in the patient’s medical history.

**Figure 2:**
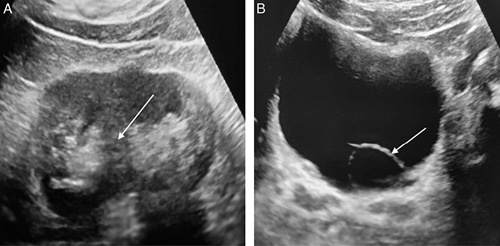
(A) Gray scale ultrasound image showing echogenic renal pelvis separated by the renal cortex in between (white arrow). No dilataion of renal pelvis seen. (B) Gray scale ultrasound image showing the cystic dilatation of intravesical part of left distal ureter (white arrow).

**Figure 3:**
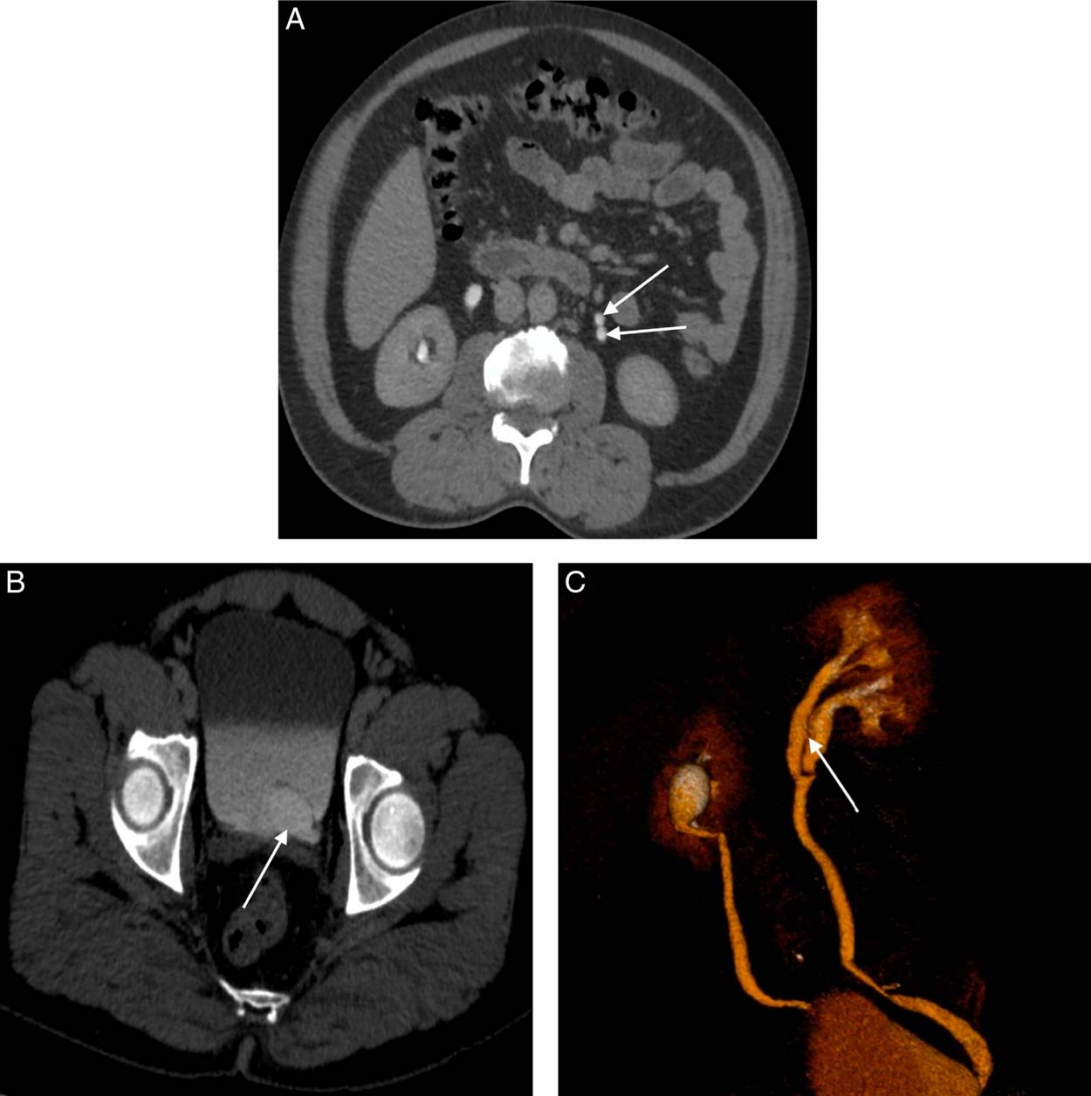
(A) Axial delayed phase contrast enhanced computed tomography (CT) image at lower level of kidney shows duplex collecting system in left side (white arrows). (B) Delayed phase axial contrast enhanced CT image at the level of pelvis shows the contrast filled outpouching (white arrow) arising from the left distal ureter. (C) 3D volume rendering technique of CT urography showing the partial duplex collecting system in left side (white arrow).

Upon physical examination no abnormalities were detected in the patient. A urine routine examination was requested, revealing 8–10 pus cells per high-power field. The results of the renal function test (RFT) were within normal limits. Abdominal and pelvic ultrasonography (USG) detected a cystic dilation of the distal part of the left ureter measuring 2.7×2.6 cm which raised concerns about a possible underlying abnormality (Fig. 2A, B). Based on the USG findings, a CT urography was conducted, which was now accessible in the rural setting, to obtain a more comprehensive evaluation of the condition. The CT urography results revealed a left intravesical ureterocele measuring 3.4×2.3 cm. Furthermore, it demonstrated a partial duplex collecting system in the left kidney, with the fusion of the upper and lower pole moiety observed at the level of the pelvic ureteric junction. Mild dilatation of the proximal ureter was also noted (Fig. 3A, B, C). He was then diagnosed with a UTI believed to be secondary to the underlying anatomical abnormality.

A urine sample was collected for culture, and the patient received medical management for an UTI with antibiotics. He was also advised to drink plenty of fluids which led to an improvement in his current symptoms. The diagnosis was explained to him, and regular follow-up visits were scheduled to monitor for symptoms and assess his RFT and USG. After a thorough discussion of the available options, the patient consented to undergo an endoscopic incision of the ureterocele. Subsequently, a cystoscope-guided puncture of the ureterocele was successfully performed without encountering any intraoperative or postoperative complications. The most recent RFT report was within the normal range. The patient has diligently attended follow-up appointments since being informed about his cause of recurrent symptoms and the need to monitor him for future symptoms and hence has expressed willingness to undergo further interventions if necessary. The patient has been symptom-free since the endoscopic incision.

## Discussion

Ureteroceles are generally accepted to be a congenital disease in pediatric populations, and in adults, some authors believe it is an acquired disease^7^. Ericson, in 1954 made the first attempt at classifying this disease^8^. It is classified into intravesical (entirely in the bladder) or ectopic (located in the bladder neck or the urethra), and ectopic is more common than intravesical ureterocele^9^. Ureterocele can arise as a potential complication of a duplicated ureter^10^. Renal duplex anomalies are common congenital anomalies that can be accurately diagnosed by prenatal sonography even when there is minimal dilatation of the renal pelvis^11^. Antenatal diagnosis allows planning of postnatal care, which may prevent UTIs and renal function impairment^11^.

Patients diagnosed with duplex ureter and ureterocele may experience a range of symptoms, including recurrent UTIs, incontinence, stone formation due to urine stasis, and episodes of acute urinary retention^12^. VUR leading to renal scarring and renal failure is also a potential complication^12^. The majority of studies have stated that most of the patients are diagnosed before they reach the age of 3 years^13^. In our case, the patient was a 41-year-old male in whom the diagnosis becomes rare because of his sex and age at diagnosis. He was born in rural Nepal, which led to no antenatal checkup and no evaluations for the symptoms in his childhood. The patient had been suffering from recurrent urinary symptoms for more than a decade. Given his current symptoms, notable history of recurrent similar episodes, and a past occurrence of urethral calculus, it became imperative to prioritize further evaluation of the urinary tract and hence an USG followed by CT urography was done.

Management of patients with ureterocele is an individualized approach^13^. It is influenced by various factors such as age, presenting complaints, presence of any reflux, functional capacity of each renal segment in case it is associated with a duplex system and complications like UTI^13^. Ureterocele in case of adults may be detected incidentally and as such does not require treatment unless it is complicated^4^. It may co-exist with other conditions such as a ureteral calculus and in these conditions it can be managed endoscopically^4^. In case of simple duplex systems where there is absence of dilatation of either moiety, with no associated obstruction or when there is no reflux, no intervention is required^11^. This patient was treated for the ongoing infection and was assessed periodically to monitor his renal structure and function. The CT urography findings of this patient were not suggestive of significant VUR or calculi and hence did not warrant any immediate measures to be taken.

While a straightforward radiological investigation and diagnosis may not appear to be a significant obstacle, it is important to note that in the rural areas of Nepal are home to many people to whom such treatment sounds like a luxury. Consequently, this frequently leads to prolonged suffering to the patient and such unusual presentations to the clinicians who finally get to evaluate them. This patient did not undergo any drastic or operative interventions for treatment. Nevertheless, the establishment of a definitive diagnosis following an extended period of unresolved suffering has notably improved the patient’s physical, mental and social well-being. Also, the importance of a proper antenatal evaluation cannot be overemphasized. While there has been notable improvement in antenatal care clinic visits over the past few decades, thanks to initiatives such as safe motherhood programs, many women still face limited access to these services. This study also highlights the importance of proper and timely antenatal evaluation not only to promote safe delivery but also to prevent long-term morbidity due to potentially correctable conditions.

## Conclusion

The case study highlights the challenges of diagnosing and managing a partial duplex collecting system with ureterocele in an adult male patient. The delayed diagnosis due to limited access to antenatal care and evaluations underscores the importance of early detection and proper antenatal evaluation to prevent long-term morbidity. While the patient did not require urgent intervention, the establishment of a definitive diagnosis and a minimally invasive procedure has significantly improved his physical, mental, and social well-being. It emphasizes the need for individualized management approaches considering factors such as age, presenting complaints, reflux, and associated complications. This case also sheds light on the impact of resource limitations in rural settings, necessitating improved access to diagnostic tools and interventions for better patient outcomes.

## Ethical approval

Ethical approval is not required for case reports in my institution (Patan Academy of Health Sciences, Bagmati Lalitpur) so ethical approval was exempted.

## Consent

Written informed consent was obtained from the patient for the publication of this case report and accompanying images. A copy of written consent is available for review by the Editor-in-Chief of this journal on request.

## Sources of funding

Not applicable.

## Author contribution

S.K.: conceptualization, mentor, and reviewer for this case report and for data interpretation; A.G.: contributed in performing literature review, writing the paper, and editing; K.S.: contributed in writing the paper; R.K.: contributed in writing the paper; S.A.: contributed in writing the paper. All authors have read and approved the manuscript.

## Conflicts of interest disclosure

All the authors declare that they have no competing interest.

## Research registration unique identifying number (UIN)

1. Name of the registry: not applicable.
2. Unique identifying number or registration ID: not applicable.
3. Hyperlink to your specific registration (must be publicly accessible and will be checked): not applicable.

## Guarantor

Shailendra Katwal.

## Provenence and peer review

Non commissioned, externally peer reviewed.

## Data availability statement

Not applicable.
